# Optimizing the Reliability and Performance of Service Composition Applications with Fault Tolerance in Wireless Sensor Networks

**DOI:** 10.3390/s151128193

**Published:** 2015-11-06

**Authors:** Zhao Wu, Naixue Xiong, Yannong Huang, Degang Xu, Chunyang Hu

**Affiliations:** 1 School of Mathematics and Computer Science, Hubei University of Arts and Science, Xiangyang 441053, China; E-Mails: wuzhao73@163.com (Z.W.); yannonghuang@gmail.com (Y.H.); pcxinx@163.com (D.X.); huchunyang@gmail.com (C.H.); 2 School of Computer Science, Colorado Technical University, Colorado Springs, CO 80907, USA

**Keywords:** wireless sensor networks, services composition, reliability optimization, performance optimization, fault tolerance

## Abstract

The services composition technology provides flexible methods for building service composition applications (SCAs) in wireless sensor networks (WSNs). The high reliability and high performance of SCAs help services composition technology promote the practical application of WSNs. The optimization methods for reliability and performance used for traditional software systems are mostly based on the instantiations of software components, which are inapplicable and inefficient in the ever-changing SCAs in WSNs. In this paper, we consider the SCAs with fault tolerance in WSNs. Based on a Universal Generating Function (UGF) we propose a reliability and performance model of SCAs in WSNs, which generalizes a redundancy optimization problem to a multi-state system. Based on this model, an efficient optimization algorithm for reliability and performance of SCAs in WSNs is developed based on a Genetic Algorithm (GA) to find the optimal structure of SCAs with fault-tolerance in WSNs. In order to examine the feasibility of our algorithm, we have evaluated the performance. Furthermore, the interrelationships between the reliability, performance and cost are investigated. In addition, a distinct approach to determine the most suitable parameters in the suggested algorithm is proposed.

## 1. Introduction

Wireless Sensor Networks (WSNs) are validated as an integral part of the Internet of Things where they extend the Internet to the physical world [1,2]. Due to their low-power, low-cost and small form factor, WSNs are widely used in Enterprise-IT systems. In order to quickly and flexibly respond to market changes, it is important that the WSN-based Enterprise-IT systems should be able to better adapt the business processes and the underlying software infrastructure [3]. To achieve this goal, organizations have focused on modeling, analysis and adaptation of business processes since early 2004 [4]. Yet, while Service-Oriented Architecture (SOA) is prospering in Enterprise-IT, WSNs have—despite contrary prognoses—largely not found their way into enterprises.

Parallel to the development of SOA, WSNs are envisioned to become an integral part of the Future Internet where they extend the Internet to the physical world. In recent years, some approaches have presented for the seamless integration WSNs with existing, widely deployed SOA technologies such as XML, Web Services, and Business Process Execution Language (BPEL) to build SCAs in WSNs [5,6]. These research results lay the groundwork for a new class of applications where all kinds of devices ranging from simple sensor nodes (SNs) to large-scale application servers interact to drive business processes in ways not possible before. In this scenario, the datastream from WSNs will influence the control flow of business processes in real-time or even trigger some business processes. In these approaches, the entire WSN or every SN can be packaged as some WSN services subject to a Web services technical standard, which can be published, located, and invoked across the Web [7]. Thus, these WSN services can be combined into the workflows in SCAs to fulfill some specific tasks in a services composition way [8,9].

From the perspective of system structure, the SCAs in WSNs are a kind of abstract of the distributed software system based on WSNs and running on the Internet. Since WSNs and the Internet are open, dynamic and difficult to control, the SCAs in WSNs have many differences from traditional software systems, for example system structures, operation mechanisms, correctness guarantees, development methods and life cycle. The traditional software systems have some characteristics, such as finite autonomy, fixed encapsulation, monotonic interaction, tightly coupled structure, and offline evolution, because of their static, closed and controllable running environment. Different from the traditional software systems, the WSN services exist in each SN in the form of active software services. Runtime SCAs in WSNs have some new characteristics that differ from those of traditional software systems, for example flexible evolution, continuous reaction and multi-target self-adaption.

**Figure 1:**
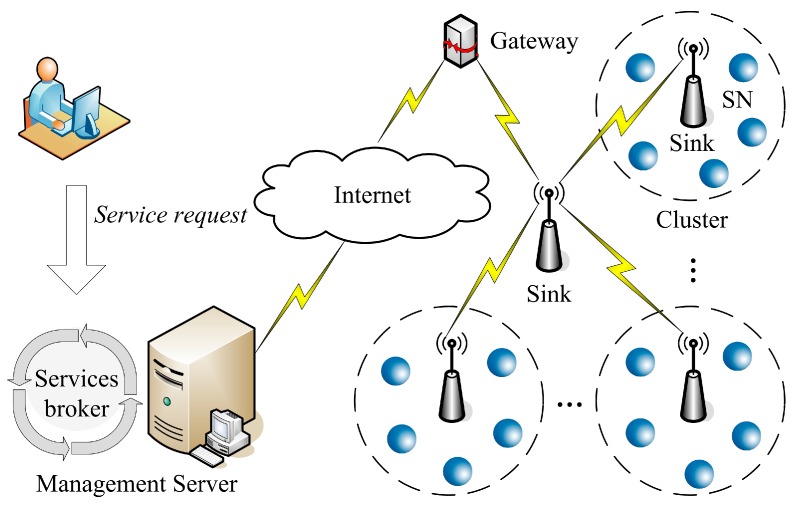
Architecture of a WSN service system.

**Figure 2:**
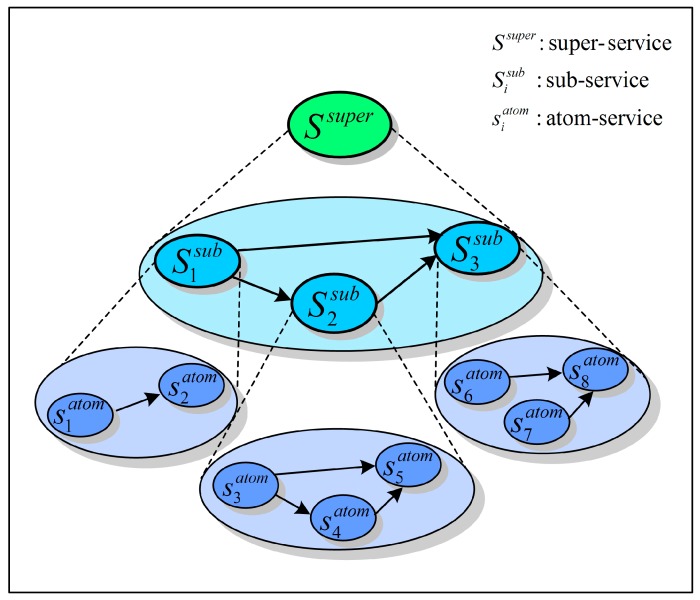
Mapping procedure from a service request to a SCA in WSNs by the SB.

These new characteristics are real challenges faced by researchers attempting to optimize the reliability and performance of SCAs in WSNs [10]. The architecture of WSN service systems with fault tolerance (FT) is considered in this paper, which is shown in Figure 1. As the data resource access and control center in the framework of WSN service systems, the WSN services broker (SB) is closely related to the reliability and performance of system [11,12]. The SB is deployed in the management server to play some important roles. To be specific, the SB manages user’s service requirements, and dynamically controls the startup, access and sharing of data resources. When a service request is received, the SB maps it into a super-service which is a logical service in a business logic layer, not a physical WSN service in the physical layer. Then, the SB divides this super-service into some sub-services according to the business rules received from the domain experts. Each sub-service represents a certain business operation in the business flow. However, in a real application scenario there are usually no physical WSN services matching these sub-services in the WSN service system. Therefore, each sub-service must be fulfilled by a services composition composing a set of physical WSN services, named atom-services (ASs). By way of collaboration among these ASs, the user’s service request can be fulfilled. The above mapping procedure from a service request to a SCA in WSNs is illustrated in Figure 2.

During the execution of a SCA in a WSN, the execution route and the selection of ASs are dynamically determined by the SB according to the running state. In addition, the outside SNs can be dynamically added in a WSN at any time. According to the business flows specification of user’s service requests as well as some business rules, the ASs corresponding to some of these new SNs may be selected to combine into the SCA during runtime by using the late binding mechanism in services composition technology [13,14]. Therefore, the software model of a SCA in WSNs is a dynamic variable. We cannot clearly know what ASs are in a SCA, as well as their running states and performance indices, until the end of the software running. However, the optimization methods for reliability and performance are essential different between the SCAs in WSNs and traditional software, so the optimization methods used for the traditional software are inapplicable to the SCAs in WSNs.

Besides the applicability of optimization methods, the computational complexity is another crucial problem. A great number of possible solutions will be evaluated in solving optimization problems for the reliability and performance of SCAs in WSNs due to the dynamic variability of software models. The traditional reliability assessment methods, such as Boolean Models, Markov Process and Monte-Carlo simulation technique, have some disadvantages. They are either only suitable for small-scale systems, or too time-consuming in simulation [15]. Different from the optimization methods for reliability and performance used for the traditional software, ones used for the SCAs in WSNs pay more attention to the flexible measure, deduce and adoption mechanism of reliability and performance based on summative evaluation on the operation information in an open running environment [16,17].

In addition to the above differences, the SCAs in WSNs are faced with the ever-changing user requests, so they must have the ability to apperceive any changes in the outside environment, and dynamically evolve to adapt to these changes. In order to provide better reliability and performance to users, the SCAs in WSNs must have more adaptability to collect various changes in real-time, to adjust themselves online in runtime [18,19].

At present, the research on the reliability and performance optimization for SCAs in WSNs is just beginning. In the face of urgent demands for SCAs with high-reliability and high-performance in WSNs in many fields, such as military affairs, precision agriculture, safety monitoring, and environmental monitoring, reliability and performance optimization has become the key to encourage the successful development, application and popularization of SCAs in WSNs [20,21].

Facing the above challenges, this paper researches the reliability and performance model of SCAs in WSNs. Based on this, an efficient optimization algorithm for reliability and performance of SCAs in WSNs is presented based on UGF and GA. The rest of this paper is organized as follows: firstly the reliability and performance model of SCAs in WSNs is presented in Section 2. Secondly, the formal definitions for the reliability and performance of SCAs in WSNs are proposed based on UGF in Section 3. On this basis, an efficient optimization algorithm for reliability and performance of SCAs in WSNs is presented based on UGF and GA in Section 4. Following this, in order to illustrate our approach, some numerical examples and analysis process are described in Section 5. Finally, the conclusions and future work are given in Section 6.

## 2. Reliability and Performance Model for SCAs in WSNs

Since the service time can take different values, the SCAs in WSNs should be considered as a multi-state system (MSS) [22] with performance depending on combination of states of its elements. In other words, the SCAs in WSNs can have different performance levels corresponding to different combinations of available and failed SNs with different processing speeds and failure rates, as well as their communication channels with different data transmission speeds and failure rates. This paper uses MSS theory to model and analyze the SCAs in WSNs. The next section briefly introduces the MSS theory.

Many real-world systems are composed of multi-state components, which have different performance levels and several failure modes with various effects on the system’s entire performance. Such systems are called MSS. The MSS was introduced in the middle of the 1970’s in [23]. The MSS can perform their tasks with various distinguished levels of efficiency usually referred to as performance rates. In other words, the MSS can have a finite number of performance rates [24]. Since the SCAs in WSNs consist of different ASs, and have a cumulative effect on the entire system performance, it can be considered as a MSS.

The reliability and performance analysis of the SCAs with fault tolerance in WSNs relates to systems for which one cannot formulate an “all or nothing” type of failure criterion [25]. The SCAs with fault tolerance in WSNs are able to perform their task with partial performance (intensity of the task accomplishment). Failures of some system elements, such as some ASs in SCAs or some SNs in WSNs, lead only to the degradation of the system performance [26,27]. In order to model and analyze the SCAs in WSNs, we use MSS theory to define their reliability and performance, which is described in the next section.

### 2.1. Reliability and Performance Definitions for SCAs in WSNs

The MSS behavior is characterized by its evolution in the space of states. Therefore, MSS reliability can be defined as its ability to remain in the acceptable state during the operation period. Since the system functioning is characterized by its output performance *G*(*t*) where *t* is time, the state acceptability depends on the value of this index. In some cases this dependency can be expressed by the acceptability function *F*(*G*(*t*)) that takes non-negative values if and only if the MSS functioning is acceptable. This takes place when the efficiency of the system functioning is completely determined by its internal state.

Much more frequently, the system state acceptability depends on the relation between the MSS performance and the desired level of this performance (demand) that is determined outside of the system. In general, the demand *W*(*t*) is also a random process. It can take discrete values from the set ***w*** = {*w*_1_, …, *w_M_*}, which is a vector of user’s requirement rates *w_j_*, (*j* = 1, …, *M*). The desired relation between the system performance and the demand can also be expressed by the acceptability function *F*(*G*(*t*),*W*(*t*)). The acceptable system states correspond to *F*(*G*(*t*),*W*(*t*)) ≥ 0, and the unacceptable states correspond to *F*(*G*(*t*),*W*(*t*)) < 0. The last inequality defines the MSS failure criterion. In many practical cases, the MSS performance should exceed the demand. In such cases the acceptability function takes the form:

(1) F(G(t),W(t))=G(t)−W(t)

From the aspect of users, the reliability of SCAs in WSNs can be defined as the probability that its performance rates satisfy user’s requirements which is described as a vector pairs (***w***,***q***). Furthermore, ***q*** = {*q*_1_, *q*_2_, …, *q_M_*} is the vector of steady state probability *q_j_* = Pr{*W* = *w_j_*}, (*j* = 1, …, *M*) according to a certain user’s requirement rate, where *W* is a random variable that represents the performance rates of SCAs in WSNs. Based on the above definition, the reliability function of SCAs in WSNs under steady state can be defined as:

(2) $$R\left(t\right)=\text{Pr}\left\{T_{f}\geq t|F\left(G\left(0\right),W\left(0\right)\right)\geq 0\right\}$$

where *T_f_* is time to failure which is the time from the beginning of the system life up to the instant when the system enters the subset of unacceptable states the first time. Therefore, the reliability function *R*(*t*) is the probability that *T_f_* is greater than or equal to the value *t* (*t* > 0), where in the initial state (at instant *t* = 0) MSS is in one of the acceptable states. Then, the reliability function *R*(*t*) under transient state can be defined as:

(3) R(t)=Pr{F(G(t),W(t))≥0}

where *G*(*t*) is the integral performance rates of SCAs in WSNs. In the interval [0, *T*], the reliability function *R_T_* of SCAs in WSNs can be defined as:

(4) $$R_{T}=\frac{1}{T}\int_{0}^{T}1\left(F\left(G\left(t\right),W\left(t\right)\right)\geq 0\right)dt$$

Based on Equation (4), it can be seen that for the discrete random demand with PMF ***w*** = {*w*_1_, …, *w_M_*}, ***q*** = {*q*_1_, …, *q_M_*}, the reliability function of SCAs in WSNs under dynamically changing user’s requirements can be defined as:

(5) $$\begin{array}{l} R\left(w,q\right)=\sum_{m=1}^{M}R\left(w_{m}\right)q_{m}\\ \;=\sum_{m=1}^{M}q_{m}\sum_{k=1}^{K}p_{k}1\left(F\left(g_{k},w_{m}\right)\geq 0\right) \end{array}$$

According to Equation (5), the reliability and performance of SCAs in WSNs can be calculated based on the probability distribution of performance rates of component services, for example sub-services and ASs shown in Figure 2. In order to calculate the reliability and performance of SCAs in WSNs, we present the probability distribution representation of performance rates for any component service, which is described in the next section.

### 2.2. Probability Distribution of Performance Rates for Any Component Service

According to its performance rates, the component service *j* within a SCA in WSNs can be of *k_j_* kinds of various states, described by $g_{j}=\{g_{j1},g_{j2},\ldots ,g_{jk_{j}}\}$, where *g_ji_* is the performance rate of component service *j* under the state *i*, *i* ∈ {1, 2, ..., *k_j_*}. Corresponding to the component service *j*, the performance rate *G_j_*(*t*) in any time *t* ≥ 0 is a random variable that gets the value from ***g****_j_*: *G_j_*(*t*) ∈ ***g****_j_*. The probability of performance rates of the component service *j* under various states in any time *t* can be described as a set $p_{j}(t)=\{p_{j1}(t),g_{j2}(t),\ldots ,g_{jk_{j}}(t)\}$, where *p_ji_*(*t*) = Pr{*G_j_*(*t*) = *g_ji_*}. Because the component service *j* is in only one of *k_j_* kinds of various states in any time *t*, these states form a mutual exclusion events complete set. Therefore, Equation (6) is satisfied:

(6) $$\sum_{i=1}^{k_{j}}p_{ji}\left(t\right)=1,\left(0\leq t\leq T\right)$$

In the end, the set of value pairs <*g_ji_*, *p_ji_*(*t*)> completely determines the probability distribution of performance rates corresponding to the component service *j* in any time *t*. Having the probability distribution of performance rates of all component services, the reliability and performance of the entire SCA can be calculated according to the composite structure by mapping the performance rates space of component services into that of the entire SCA. In order to achieve this mapping, the structure functions of performance rates are defined in the next section.

### 2.3. Structure Function of Performance Rates for SCAs in WSNs

The structure function of SCAs in WSNs can be defined as follows. Let ***L****^n^* be the possible combinations of performance rates of all component services, and ***M*** = {*g*_1_, …, *g_k_*} be the possible values range of performance rates of SCAs in WSNs. ***L****^n^* can be defined as:

(7) $$L^{n}=\{g_{11},\ldots g_{1k_{1}}\}\times \{g_{21},\ldots g_{2k_{2}}\}\times \ldots \times \{g_{n1},\ldots g_{nk_{n}}\}$$

For a SCA consisting of *n* ASs, the performance rates of the ASs unambiguously determine the performance rates of the SCA. These ASs have certain performance rates corresponding to their states in every moment. The states of this ASs determine that of the SCA. Assume that the SCA has *K* different states and that *g_i_* is the SCA performance rate in state *i* (*i* ∈ {0, ···, *K*−1}). The SCA performance rate is a random variable that takes values from the set {*g*_1_, ···, *g_K_*_−1_}. Then, the transform function ϕ(*G*_1_(*t*), …, *G_n_*(*t*)): ***L****^n^*→***M***, called as structure function, can map the performance rates space of component services into that of the entire SCA. Hence, the reliability model of SCAs in WSNs can be defined as ***g****_j_*, ***p****_j_*(*t*), 1 ≤ *j* ≤ *n*, ϕ(*G*_1_(*t*), …, *G_n_*(*t*)).

The structure function of SCAs in WSNs establishes a feasible way to calculate the reliability and performance of the entire SCA using those of component services. In order to efficiently calculate the reliability and performance by using a fast algebraic procedure, the UGF technique is introduced into our model. Based on UGF, the reliability and performance of SCAs in WSNs are defined in the next section.

## 3. Reliability and Performance Definition Based on UGF

In this paper, we choose the UGF technique to achieve high efficiency in calculating the reliability and performance of SCAs. The next section gives the reasons for selecting it.

### 3.1. Advantage of UGF Technique

In general, the methods of MSS reliability assessment are based on four different approaches [15]: (1) an extension of the Boolean models [28] to the multi-valued case; (2) the stochastic process (mainly Markov [29] and semi-Markov) approach; (3) the Monte-Carlo simulation technique [30]; (4) the UGF approach [31].

The approach based on the extension of Boolean models is historically the first method that was developed and applied for the MSS reliability evaluation. It is based on the natural expansion of the Boolean methods to the multi-state systems.

The stochastic process methods that are widely used for the MSS reliability analysis are more universal. The methods can be applied only to relatively small MSSs because the number of system states increases dramatically with the increase in the number of system elements.

Even though almost every real world MSS can be represented by the Monte-Carlo simulation for the reliability assessment, the main disadvantages of this approach are the time and expenses involved in the development and execution of the model.

The computational burden is the crucial factor when one solves optimization problems where the reliability measures have to be evaluated for a great number of possible solutions along the search process. This makes the use of the first three methods have a problem in reliability optimization [32]. On the contrary, the UGF allows one to find the entire MSS performance distribution based on the performance distribution of its elements by using a fast algebraic procedure. The analysts can use the same recursive procedures for MSS with a different physical nature of performance and different types of element interaction [33]. Therefore, it is fast enough for dynamically changing SCAs in WSNs.

The UGF generalizes the well-known ordinary generating function. Its basic ideas were introduced by Ushakov [34]. It has proved very convenient for numerical realization [35]. In addition, it requires relatively small computational resources for evaluating MSS reliability and performance indices. The advantages of UGF were analyzed in detail in [36], as well as its computational complexity. The efficiency of UGF was discussed in [37]. It has proved more accurate and efficient. Therefore, it can be used in complexes reliability and performance optimization problems. Because the relationships between the system state probability and the system output performance rates can be expressed definitely by UGF, and the UGF of system can be obtained by calculating those of components simply, UGF has proved to be an efficient reliability and performance assessment approach that is suitable to various MSS.

The problem of system reliability and performance analysis usually includes evaluation of the probability mass function (PMF) of some random values characterizing the system’s behavior. These values can be very complex functions of a large number of random variables. The explicit derivation of such functions is an extremely complicated task. Fortunately, the UGF method for many types of system allows one to obtain the system u-function recursively. This property of the UGF method is based on the associative property of many functions used in reliability engineering. The recursive approach presumes obtaining u-functions of subsystems containing several basic elements, and then treating the subsystem as a single element with the u-function obtained when computing the u-function of a higher level subsystem. Combining the recursive approach with the simplification technique reduces the number of terms in the intermediate u-functions, and provides a drastic reduction of the computational burden.

For the above reasons, we selected UGF technique to develop an efficient reliability and performance evaluation method for SCAs in WSNs. In order to express the u-functions of reliability and performance of SCAs in WSNs, their UGF definitions are proposed in the next section.

### 3.2. Reliability and Performance Definitions of SCAs in WSNs Based on UGF

Based on the reliability and performance model presented in Section 2, the u-function of reliability of SCAs in WSNs can be defined according to [24]. The general form of definition as follows:

The reliability of the entire SCA (or a component service within a SCA) in WSNs is a random variable *X*. According to the UGF technique, the probability distribution of performance can be obtained using a formal operator *z* that resembles the procedure of the product of polynomials. Therefore, its u-function can be defined as:

(8) $$u\left(z\right)=\sum_{k=1}^{K}p_{k}{}^{\circ}z^{X_{k}}$$

where the discrete variable *X* has *K* possible values, *p_k_* is the reliability when *X* is in the performance state *X_k_*. Based on this definition, the u-function of the reliability of the entire SCA (or one of its component services) in transient state can be expressed as:

(9) $$U\left(t,z\right)=\sum_{k=1}^{K}p_{k}\left(t\right)\cdot z^{G_{k}}$$

Because *U*(*z*) relates the performance rates *G_k_* with its state probabilities *p_k_*, it describes the probability distribution of reliability of SCAs (or a component service) in WSNs. Following this, in order to express other indices related to reliability, such as availability, output performance and unfinished performance, we define three performance operators based on the above u-function of reliability.

(1) Availability operator δ*_A_*: The availability operator δ*_A_* is defined as the sum of all probabilities of system states satisfying the condition *F*(*G_k_*, *W_m_*) ≥ 0. It can be formulated as:

(10) $$\begin{array}{cc} \delta_{A}\left(U\left(z\right),F,W_{m}\right) & \text{=}\delta_{A}\left(\sum_{k=1}^{K}p_{k}\cdot z^{G_{k}},F,W_{m}\right)\\ & =\sum_{k=1}^{K}p_{k}\cdot 1\left(F\left(G_{k},W_{m}\right)\geq 0\right) \end{array}$$

(2) Output performance operator δ*_G_*: The output performance operator δ*_G_* is defined as the sum total of the products of each performance rate *G_k_* and its corresponding state probability *p_k_*. It can be formulated as: ${\text{δ}}_{G}\left(U(\text{z})\right)=\sum_{k=1}^{K}p_{k}\cdot G_{k}$.

(3) Unfinished performance operator *δ_U_*: The unfinished performance operator δ*_U_* is defined as the sum total of the products of un-acceptability (*i.e.*, unfinished performance max{−F(*G_k_*, *W_m_*), 0}) and its corresponding state probability *p_k_*. It can be formulated as:

(11) $$\begin{array}{cc} {\text{δ}}_{U}\left(U\left(z\right),F,W_{m}\right) & ={\text{δ}}_{U}\left(\sum_{k=1}^{K}p_{k}\cdot z^{G_{k}},F,W_{m}\right)\\ & =\sum_{k=1}^{K}p_{k}\cdot \max\left\{-F\left(G_{k},W_{m}\right),0\right\} \end{array}$$

Based on the above performance operators, the three indices related reliability for SCAs (or a component service) in WSNs can be defined as follows:

(1) Availability: The availability is a prediction about the ability of a SCA to perform its designated function with required performance. It is defined as the sum total of the products of the steady state probability *q_m_* and its corresponding probability satisfying the condition *F*(*G_k_*, *W_m_*) ≥ 0, *i.e.*, δ*_A_*(*U*(*z*), *F*, *W_m_*). It can be formulated as:

(12) $$E_{A}=E_{A}\left(W,q\right)=\sum_{m=1}^{M}q_{m}\cdot {\text{δ}}_{A}\left(U\left(z\right),F,W_{m}\right)=\sum_{m=1}^{M}q_{m}\left(\sum_{k=1}^{K}p_{k}\cdot 1\left(F\left(G_{k},W_{m}\right)\geq 0\right)\right)$$

(2) Output performance expectation: The output performance expectation is a prediction about the quality of a future task-related behavior by a SCA in WSNs. It is defined the sum total of the products of each performance rate and its corresponding state probability. It can be calculated by the output performance operator δ*_G_*:

(13) $$E_{\text{G}}={\text{δ}}_{G}\left(U(\text{z})\right)=\sum_{k=1}^{K}p_{k}\cdot G_{k}$$

(3) Unfinished performance requirement: The unfinished performance requirement is a prediction about the risk of a SCA to perform its designated function without required performance. It is defined as the sum total of the products of the steady state probability *q_m_* and its corresponding probability unsatisfying the condition *F*(*G_k_*, *W_m_*) ≥ 0, *i.e.*, δ*_U_*(*U*(*z*), *F*, *W_m_*). It can be formulated as:

(14) $$E_{U}\left(W,q\right)=\sum_{m=1}^{M}q_{m}\cdot {\text{δ}}_{U}\left(U\left(z\right),F,W_{m}\right)$$

### 3.3. Composite Operators of Reliability and Performance Indices Based on UGF

For a component based system, the overall reliability and performance are determined by all of its components. The UGF technique provides a fast route to obtain the overall reliability and performance from that of the various components. In order to achieve this goal, some composite operators are defined according to the system structure function *f* (*X*_1_, …, *X_n_*) presented in Section 2.3. In other words, the properties of the composite operator strictly depend on the properties of the system structure function. Since the procedure of the multiplication of the probabilities in composite operators is commutative and associative, the entire operator can also possess these properties if the function possesses them.

Based on the reliability and performance definition expressed by UGF for component services in Section 3.2, the u-function composite operators Ω can be designed for various reliability and performance indices in diverse patterns of services composition. By the Ω calculation, the overall system reliability and performance can be worked out based on those of all components.

Since the procedure of the multiplication of the probabilities in composite operators is commutative and associative, two rules must be satisfied in the design of u-function composite operators Ω as follows:

(1) Commutativity rule: The commutativity rule can be formulated as follows:

(15) $$\begin{array}{l} \Omega \left(U_{1}\left(z\right),\ldots ,U_{k}\left(z\right),U_{k+1}\left(z\right),\ldots ,U_{n}\left(z\right)\right)\\ \;=\Omega \left(U_{1}\left(z\right),\ldots ,U_{k+1}\left(z\right),U_{k}\left(z\right),\ldots ,U_{n}\left(z\right)\right) \end{array}$$

(2) Associativity rule: The associativity rule can be formulated as follows:

(16) $$\begin{array}{l} \Omega \left(U_{1}\left(z\right),\ldots ,U_{k}\left(z\right),U_{k+1}\left(z\right),\ldots ,U_{n}\left(z\right)\right)\\ \;=\Omega \left(\Omega \left(U_{1}\left(z\right),\ldots ,U_{k}\left(z\right)\right),\Omega \left(U_{k+1}\left(z\right),\ldots ,U_{n}\left(z\right)\right)\right) \end{array}$$

According to the above design rules, the generic form of composite operators Ω can be expressed as:

(17) $$\Omega \left(\sum_{\forall k}p_{k}\cdot z^{G_{k}},\sum_{\forall l}p_{l}\cdot z^{G_{l}}\right)=\sum_{\forall k}\sum_{\forall l}p_{k}\cdot p_{l}\cdot z^{f\left(G_{k},G_{l}\right)}$$

where *f* (*G_k_*, *G_l_*) can be defined according to the reliability and performance indices and composition structures of the SCAs in WSNs. Based on the UGF technique mentioned above, we propose an efficient reliability and performance optimization algorithm for WSN service systems in the next section.

## 4. Reliability and Performance Optimization Algorithm for WSN Service Systems

### 4.1. Architecture of WSN Service Systems with FT

In order to assure the correctness of observed data, and improve the reliability of SCAs in WSNs, some redundant SNs are deployed in WSN service systems with FT. These redundant SNs compose some sensor clusters according to the observed objects, which is depicted in Figure 1. In other words, the SNs within the same cluster are responsible for the same observed object. From the perspective of the correctness of observed data, the redundant SNs should send the same observed data for the same observed object at the same observation time.

In the architecture of WSN service systems with FT, the SNs within the same sensor cluster are controlled by the same cluster-sink. These cluster-sinks are responsible for receiving and checking the observed data from SNs within their clusters. In order to further reduce the energy consumption, n-version programming (NVP) is introduced into the check mechanism of cluster-sinks in the suggested architecture of WSN service systems with FT. 

From Figure 1, one can see that the topology of WSN service systems with FT is a star structure. At every moment, outside SNs (ASs) can be dynamically added to a cluster according to the actual needs without requiring configuration changes. Therefore, the star structure can help WSAs meet the scalability demands adequately. The management server lies in the center of the star topology, which controls the startup, initialization, distribution and recovery of the sinks, cluster-sinks and SNs dynamically.

In this architecture, the sink is responsible for receiving the observed data from the cluster-sinks, and sending it to the management server through a gateway. From the perspective of data processing, the SNs can be considered as resources, because they provide the observed data of target objects to the WSN service system.

The SB is the entrance of a WSN service system for the service requests from users. It is responsible for the mapping from the service requests to the SCAs in WSNs. Figure 2 illustrates this mapping process by the SB, which forms a tree structure with three levels respectively representing the SCAs with different abstract granularities. The top level represents the super-service corresponding to the service request from users; the middle level represents the sub-services composition generated by way of the mapping according to the business rules; the bottom level represents the ASs composition comprising the physical SN services in WSNs.

The services composition application (SCA) in WSNs consists of a set of ASs that should be executed by resources of different types (*i.e.*, the SNs of different types). Therefore, when receiving a service request from a user the SB will allocate suitable resources, *i.e.*, SNs, for the initiatory atom-service (AS) according to the observed object and the type of SNs, and execute this AS. Other ASs require outputs from preordered AS/ASs as inputs for their execution. The order of ASs’ execution is determined by the execution logics of the SCA in WSNs. When the results are returned from an AS or some ASs, the SB transforms them into the next ASs as their inputs according to the execution logics, and allocates suitable resources to execute them. When all of the ASs within a SCA in a WSN are fulfilled and the final result is returned, the service request is completely executed. In the end, the final result will be returned to the user by the SB.

**Figure 3:**
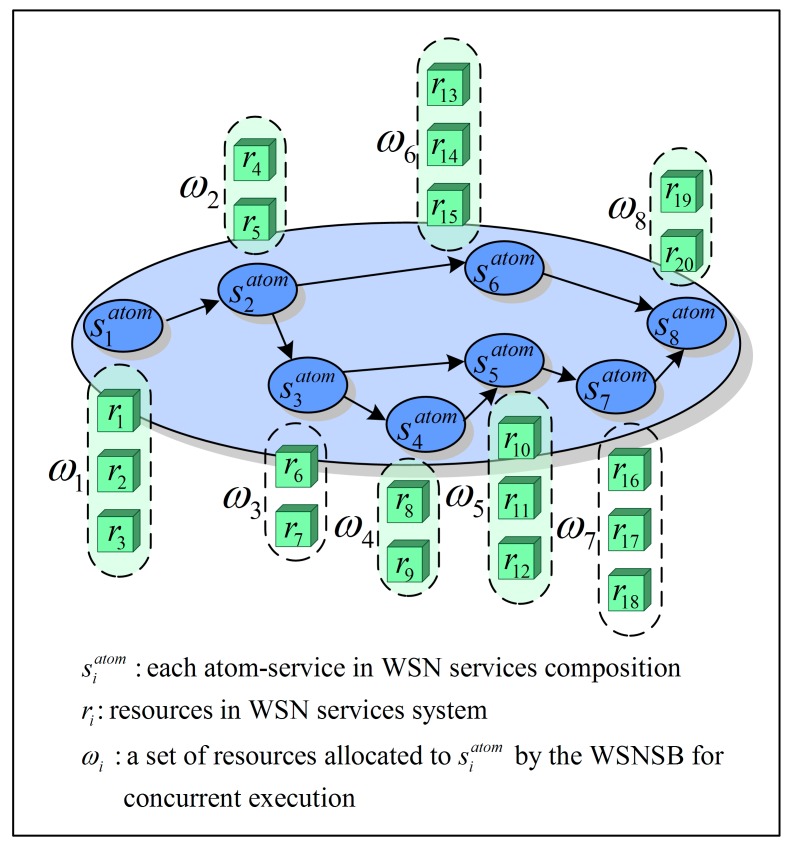
Resource allocation for ASs by the SB.

In order to simplify the complexity of the problem, we assume here each resource, *i.e.*, SN, can process only a single AS at the same time when it is available. On the other hand, the same AS can be assigned to several resources of the same type, *i.e.*, several SNs within the same sensor cluster, for parallel execution when there are multiple SNs responsible for the same observed object. Considering the reliability and efficiency, the SB usually allocates multiple SNs for each AS to execute it in parallel. For example, the ASs $s_{1}^{atom}$, $s_{2}^{atom}$, ..., $s_{8}^{atom}$ are respectively assigned to the SNs *ω*_1_ = {*r*_1_, *r*_2_, *r*_3_}, *ω*_2_ = {*r*_4_, *r*_5_}, ω_3_ = {*r*_6_, *r*_7_}, ω_4_ = {*r*_8_, *r*_9_}, ω_5_ = {*r*_10_, *r*_11_, *r*_12_}, ω_6_ = {*r*_13_, *r*_14_, *r*_15_}, ω_7_ = {*r*_16_, *r*_17_, *r*_18_}, and *ω*_8_ = {*r*_19_, *r*_20_}, which is illustrated in Figure 3. In order to improve the reliability of WSN service systems, a FT model is introduced in the suggested architecture. When the first correct result corresponding to an AS is returned from one of the allocated SNs, the SB will make a mark for the finished AS, and cancel the execution of other SNs allocated to this AS. The detailed FT model and FT mechanism are proposed in the next section.

### 4.2. FT Model in WSNs Service System

For the convenient description in the latter, this section gives some notations listed in Table 1.

**Table 1:** Notations.

Notation	Definition
*c*	cluster
*n_c_*, *B_c_*	the number of functionally equivalent SNs in cluster *c*.
*i*, *j*, *y*	No. of sensor node in a cluster.
*r_ci_*	estimated reliability of *i*-th sensor node in cluster *c*.
*τ_ci_*	constant observation time of *i*-th sensor node in cluster *c*.
*k_c_*	The cluster-sink sends one observed data to sink, if at least *k_c_* out of *n_c_* outputs agree.
*h_c_*	The total number of hardware units in cluster *c*.
*a_c_*	The availability of each hardware unit in cluster *c*.
*H_c_*	The number of hardware units available in cluster *c*.
*L_c_*	The number of SNs that can be executed simultaneously in cluster *c*.
*T_c_*	The time used for the entire cluster-sink execution.
*T*	The random task execution time used for the entire SCA.
*w*	A maximal allowed system execution time used for the entire SCA.
*F*(*T*,*w*)	The system’s acceptability function
*R*(*w*)	The system’s reliability function
$\tilde{\varepsilon }(w)$	The conditional expected system execution time
*Q_c_*(*x*)	The probabilities function of the number of SNs that can be simultaneously executed.
*t_ci_*(*l_c_*)	The termination time for the SN *i* when there are *l_c_* SNs that can run simultaneously in cluster *c*.
*m*_1_, *m*_2_, ..., $m_{n_{c}}$	The order of SNs corresponding to of their termination time.
$s_{cm_{i}}$	The random binary variable representing the success of SN *m_i_* in cluster *c*.
$u_{cm_{i}}(z)$	The PMF of the success of SN *m_i_* in cluster *c*.
$\underset{+}{⊗}$	Composition operator over u-functions
$U_{cj}(z,l_{c})$	The PMF of the number of correct outputs in cluster *c* after the execution of a group of first *j* SNs.
*π_jk_*	The probability that the group of first *j* SNs produces *k* correct outputs.
${\tilde{u}}_{c}(z,l_{c}(x))$	The u-function representing the conditional PMF *p_cj_*(*l_c_*(*x*)), $t_{cm_{j}}(l_{c}(x))$
${\tilde{U}}_{c}(z)$	The u-function representing the PMF of the random value *T_c_*.
$\tilde{U}(z)$	The u-function $\tilde{U}(z)$ representing the PMF of *T*.
$\hat{U}(z,x)$	The u-function representing the conditional PMF of the system execution time *T*
x*_c_	The permutation of *B_c_* different integer numbers ranging from 1 to *B_c_*.
***y**_c_*	The binary vector determining the subset of SNs selected for cluster *c*, ***y**_c_* = {*y_c1_*, …, $y_{cB_{c}}$}.
*ω_cb_*	The cost of SN *b* used in cluster *c*
Ω	The entire system cost
Ω*	The MAX allowable system cost

It is assumed that *n_c_* functionally equivalent SNs are available for each cluster *c* in a WSN service system with FT. Each sensor node (SN) *i* has an estimated reliability *r_ci_* and constant observation time *τ_ci_* (the time for sending and transferring data is neglected). Failures of SNs in each cluster are statistically independent, as well as the total failures of the different clusters, because each SN runs independently on different hardware units.

The check mechanism presumes that the different SNs in the same cluster send their observed data to the cluster-sink at first. Then, the cluster-sink compares received observation data with each other. The cluster-sink sends one observed data to sink, if at least *k_c_* out of *n_c_* outputs agree. Otherwise, the cluster-sink discards these received observation data and requires the SNs for next observation.

The SNs in each cluster *c* run on parallel hardware units. The total number of hardware units is *h_c_*. The hardware units are independent and identical. The availability of each hardware unit is *a_c_*. The number *H_c_* of hardware units available at the moment determines the amount of available computational resources and, therefore, the number *L_c_* of SNs that can be executed simultaneously. In other words, *L_c_* depends on *H_c_*. No hardware unit can change its state during execution.

The SNs in each cluster *c* start their execution in accordance with a predetermined order list. The *L_c_* first SNs from the list start their execution simultaneously (at time zero). If the number of terminated SNs is less than *k_c_*, after termination of each SN a new SN from the list starts its execution immediately. If the number of terminated SNs is not less than *k_c_*, after termination of each SN the cluster-sink compares their outputs. If *k_c_* outputs are identical, the cluster-sink terminates all SNs that are still executed; otherwise a new SN from the list is executed immediately.

If after termination of *n_c_* SNs the number of identical outputs is less than *k_c_*, the cluster-sink and the entire WSNs services system fail.

In the case that cluster-sink sends the observed data to the sink successfully, the time *T_c_* used for the entire cluster-sink execution is equal to the termination time of the SN that has produced the *k_c_*-th correct output (in most cases, the time needed by the cluster-sink to make the decision can be neglected). It can be seen that the cluster-sink execution time is a random variable depending on the reliability and the time used for the SNs’ execution and on the availability of the hardware units. We assume that if the cluster-sink fails to send the observed data to sink then its execution time is equal to infinity.

The sum of the random execution time of each cluster-sink gives the random task execution time *T* for the entire SCA in WSNs. In order to estimate both the system’s reliability and its performance, different measures can be used, depending on the application.

In a WSNs service system, the execution time of each task is of critical importance. Given the fixed mission time is *T*, the system’s acceptability function is defined as *F*(*T*,*w*) = 1(*T* < *w*), where *w* is a maximal allowed system execution time. The system’s reliability *R*(*w*) = *E*(*F*(*T*,*w*)) in this case is the probability that the correct output is produced in time less than *w*. therefore, the conditional expected system execution time can be defined as:

(18) $$\tilde{\varepsilon }(w)=\frac{E(T\times 1(T<w))}{R(w)}$$

where $\tilde{\varepsilon }(w)$ is considered to be a measure of the system’s performance, which determines the SCA’s expected execution time given that the system does not fail.

In a WSN service system, the system’s average productivity (the number of executed tasks) over a fixed mission time is of interest, the system’s acceptability function is defined as *F*(*T*) = 1(*T* < *∞*), the system’s reliability is defined as the probability that it produces correct outputs regardless of the total execution time (this index can be referred to as *R*(*∞*)), and the conditional expected system execution time $\tilde{\varepsilon }(\infty )$ is considered to be a measure of the system’s performance.

Considering the above FT mechanism, the following sections discuss the approach for calculating the reliability and performance of a WSN service system.

#### 4.2.1. Determining the Number of SNs that Can Be Simultaneously Executed

The reliability and performance of a WSN service system are influenced by the number of SNs that can be executed simultaneously. This section discusses how to determine the PMF of the number of SNs that can be simultaneously executed.

The number *x* of available hardware units in cluster *c* can vary from 0 to *h_c_*. Given that all of the units are identical and have availability *a_c_*, one can easily obtain the probabilities of the number of SNs that can be simultaneously executed, *i.e.*, *Q_c_*(*x*) = Pr{*H_c_* = *x*} for 0 ≤ *x* ≤ *h_c_*:

(19) $$Q_{c}(x)=\text{Pr}\{H_{c}=x\}=\left(\begin{array}{c} h_{c}\\ x \end{array}\right){a_{c}}^{x}{\left(1-a_{c}\right)}^{h_{c}-x}$$

The number *x* of available hardware units determines the number *l_c_*(*x*) of SNs that can be executed simultaneously. Therefore:

(20) $$\text{Pr}\{L_{c}=l_{c}(x)\}=Q_{c}(x)$$

Thus, the pairs < *Q_c_*(*x*), *l_c_*(*x*)> for 0 ≤ *x* ≤ *h_c_* determine the PMF of the discrete random value *L_c_*. Having the PMF of the number of SNs that can be simultaneously executed, if the termination time of each SN can be calculated, the PMF of execution time for each SN can be determined. The next section presents the algorithm used for calculating the termination time of each sensor node.

#### 4.2.2. Determining the Termination Time of SN

In each cluster *c*, a sequence where each SN starts its execution is defined by the numbers of SNs. This means that each SN *i* starts its execution not earlier than SNs 1, …, *i*−1 and not later than SNs *i* + 1, …, *n_c_*. If the number of SNs that can run simultaneously is *l_c_* then we can assume that the SNs run on *l_c_* independent processors. Let *α_m_* be the time when processor *m* terminates the execution of a SN and is ready to run the next SN from the list of not executed SNs. Having the execution time of each SN *τ_ci_* (1 ≤ *i* ≤ *n_c_*), one can obtain the termination time *t_ci_*(*l_c_*) for each SN *i* using the following simple algorithm.

**Algorithm 1.** Calculating the termination time *t_ci_*(*l_c_*) for each SN.Assign *α*_1_ = ··· = *α_c_* = 0 (all of the units are ready to run the SNs at time 0).For *i* = 1, ···, *n_c_* repeat:
2.1.Find any *m* (1 ≤ *m* ≤ *l_c_*): *α_m_* = *min*{*α*_1_, ···, $\alpha_{l_{c}}$} (*m* is the number of the earliest processor that is ready to run a new SN from the list).2.2.Obtain *t_ci_*(*l_c_*) = *α_m_* + *τ_ci_* and assign *α_m_* = *t_ci_*(*l_c_*)

The time *t_ci_*(*l_c_*) (1 ≤ *i* ≤ *n_c_*) corresponds to the intervals between the beginning of cluster execution and the moment when the SNs produce their outputs. Observe that the SNs that start execution earlier can terminate later: *j* < *y* does not guarantee that *t_ci_*(*l_c_*) ≤ *t_cy_*(*l_c_*). In order to obtain the sequence, in which the SNs produce their outputs, the termination time should be sorted in increasing order $t_{cm_{1}}(l_{c})\leq t_{cm_{2}}(l_{c})\leq \cdots \leq t_{cm_{n_{c}}}(l_{c})$ which gives the order of SNs *m*_1_, *m*_2_, …, $m_{n_{c}}$ corresponding to of their termination time.

The ordered list *m*_1_, *m*_2_, …, $m_{n_{c}}$ determines the sequence of SN outputs in which they arrive at the cluster-sink. Now one can consider the cluster *c* as a system in which the *n_c_* SNs are executed consecutively according to the order $m_{1},m_{2},\cdots ,m_{n_{c}}$ and produce their outputs at time $t_{cm_{1}}(l_{c})$, $t_{cm_{2}}(l_{c})$, …, $t_{cm_{n_{c}}}(l_{c})$.

Based on the PMF of *L_c_*, which can be obtained by Equations (19) and (20), and the PMF of $t_{cm_{j}}(l_{c})$, which can be derived by the algorithm in this section, the PMF of execution time for each SN can be determined. This provides a way to calculate the reliability and performance for each cluster and the entire system, which is presented in the next section.

#### 4.2.3. Determining the Reliability and Performance of Each Cluster and the Entire System

Let $r_{cm_{i}}$ be the reliability of the SN that produces *i*-th output in cluster *c*. In other words, $r_{cm_{i}}$ is equal to the probability that this output is correct. Consider the probability that *k* out of *n* first SNs of cluster *c* succeed. Thus, this probability can be obtained as:

(21) $$R_{k}=\left[\prod_{i=1}^{n}\left(1-r_{cm_{i}}\right)\right]\left[\sum_{i_{1}=1}^{n-k+1}\frac{r_{cm_{i_{1}}}}{1-r_{cm_{i_{1}}}}\sum_{i_{2}=i_{1}+1}^{n-k+2}\frac{r_{cm_{i_{2}}}}{1-r_{cm_{i_{2}}}}\cdots \sum_{i_{k}=i_{k-1}+1}^{n}\frac{r_{cm_{i_{k}}}}{1-r_{cm_{i_{k}}}}\right]$$

The cluster *c* produces the correct output directly after the end of the execution of *j* SNs (*j* ≥ *k_c_*) if the *m_j_*-th SN succeeds and exactly *k_c_* − 1 out of the first executed *j* − 1 SNs succeed. Thus, the probability of such event *p_cj_*(*l_c_*) is:

(22) $$p_{cj}(l_{c})=r_{cm_{j}}\left[\prod_{i=1}^{j-1}\left(1-r_{cm_{i}}\right)\right]\left[\sum_{i_{1}=1}^{n-k_{c}+1}\frac{r_{cm_{i_{1}}}}{1-r_{cm_{i_{1}}}}\sum_{i_{2}=i_{1}+1}^{n-k_{c}+2}\frac{r_{cm_{i_{2}}}}{1-r_{cm_{i_{2}}}}\cdots \sum_{i_{k_{c}}-1=i_{k_{c}-2}+1}^{j-1}\frac{r_{cm_{i_{k_{c}-1}}}}{1-r_{cm_{i_{k_{c}-1}}}}\right]$$

Observe that *p_cj_*(*l_c_*) is the conditional probability that the cluster execution time is $t_{cm_{j}}(l_{c})$ given *l_c_* SNs can be executed simultaneously, which can be formulated as follows:

(23) $$p_{cj}(l_{c})=\text{Pr}\{T_{c}=t_{cm_{j}}(l_{c})|L_{c}=l_{c}\}$$

Having the PMF of *L_c_* we can now obtain for 1 ≤ *x* ≤ *h_c_*:

(24) $$\begin{array}{l} \text{Pr}\{T_{c}=t_{cm_{j}}(l_{c}(x))\}\\ =\text{Pr}\{T_{c}=t_{ck_{j}}(l_{c}(x))|L_{c}=l_{c}(x)\}\text{Pr}\{L_{c}=l_{c}(x)\}\\ =p_{cj}(l_{c}(x))Q_{c}(x) \end{array}$$

Thus, the pairs $<t_{cm_{j}}(l_{c}(x)),p_{cj}(l_{c}(x))Q_{c}(x)>$, obtained for 1 ≤ *x* ≤ *h_c_* and *k_c_* ≤ *j* ≤ *n_c_*, determine the PMF of SN execution time *T_c_*.

Since the events of successful cluster execution termination for different *j* and *x* are mutually exclusive, we can express the probability of cluster *c* success as:

(25) $$R_{c}(\infty )=\text{Pr}\{T_{c}<\infty \}=\sum_{x=1}^{h_{c}}\left[Q_{c}(x)\sum_{j=k_{c}}^{n_{c}}p_{cj}(l_{c}(x))\right]$$

Since failure of any cluster constitutes the failure of the entire system, the system’s reliability can be expressed as:

(26) $$R(\infty )=\prod_{c=1}^{C}R_{c}(\infty )$$

From the PMF of execution time *T_c_* for each cluster *c*, one can obtain the PMF of the execution time of the entire system, which is equal to the sum of the execution time of clusters:

(27) $$T=\sum_{c=1}^{C}T_{c}$$

Having the PMF of the execution time of the entire system, we can evaluate the reliability and performance of a SCA in a WSN based on UGF. On this basis, we can embed this evaluation algorithm in a GA framework for optimizing the system reliability and performance. The optimization of the reliability and performance for SCAs in WSNs based on the UGF technique and GA framework is proposed in the next section.

### 4.3. Optimizing the Reliability and Performance for SCAs in WSNs

#### 4.3.1. Evaluating the Execution Time Distribution of Clusters

In order to obtain the execution time distribution for a cluster *c* for a given *l_c_* in the form *p_cj_*(*l_c_*), $t_{cm_{j}}(l_{c})$ (*k_c_* ≤ *j* ≤ *n_c_*), one can determine the realizations $t_{cm_{j}}(l_{c})$ of the execution time *T_c_*(*l_c_*) using the Algorithm 1 presented in Section 4.2.2 and the corresponding probabilities *p_cj_*(*l_c_*) using Equation (26). However, the probabilities *p_cj_*(*l_c_*) can be obtained in a much simpler way using Algorithm 2 based on the UGF technique which allows one to find the performance distribution of the entire system based on ones of its elements by using a fast algebraic procedure.

**Algorithm 2.** Evaluating the probability distribution *p_cj_*(*l_c_*) of execution time of clusters.For the given *l_c_*, determine the order of sensor node termination *m*_1_, *m*_2_, …, $m_{n_{c}}$ using AlgorithmDetermine the u-function of each sensor node of cluster *c* according to Equation (24).Define *U_c_*_0_(*z*, *l_c_*) = 1. For *k* < *k_c_* − *n_c_* + *j*:3.1.Obtain *U_cj_*(*z*, *l_c_*) using Equation (26) and, after collecting like terms, represent it in the form of Equation (27).3.2.Remove *U_cj_*(*z*, *l_c_*) from all the terms *π_jk_z^k^* For which *j* = 1, 2, ···, *n_c_*.3.3.If *j* ≥ *k_c_*, assign: *p_cj_*(*l_c_*) = ${\text{π}}_{jk_{c}}$ and remove term ${\text{π}}_{jk_{c}}z^{k_{c}}$ from *U_cj_*(*z*, *l_c_*).

Let the random binary variable $s_{cm_{i}}$ be an indicator of the success of SN *m_i_* in cluster *c*, such that $s_{cm_{i}}$ = 1 if the SN produces the correct output; and $s_{cm_{i}}$ = 0 if it produces the wrong output. The PMF of $s_{cm_{i}}$ can be represented by the following u-function:

(28) $$u_{cm_{i}}(z)=r_{cm_{i}}z^{1}+(1-r_{cm_{i}})z^{0}$$

It can be easily seen that using the operator $\underset{+}{⊗}$ we can obtain the u-function:

(29) $$U_{cj}(z,l_{c})=\underset{+}{⊗}\left(u_{cm_{i}}(z),\cdots ,u_{cm_{j}}(z)\right)$$

that represents the PMF of the number of correct outputs in cluster *c* after the execution of a group of first *j* SNs (the order of elements *m*_1_, *m*_2_, …, $m_{n_{c}}$ and, therefore, *U_cj_*(*z*, *l_c_*) depend on *l_c_*). Indeed, the resulting polynomial relates the probabilities of combinations of correct and wrong outputs (the product of the corresponding probabilities) with the number of correct outputs in these combinations (the sum of success indicators). Observe that after collecting the like terms (corresponding to obtaining the overall probability of a different combination with the same number of correct outputs) *U_cj_*(*z*, *l_c_*) takes the form:

(30) $$U_{cj}(z,l_{c})=\sum_{k=0}^{j}\pi_{jk}z^{k}$$

where *π_jk_* is the probability that the group of first *j* SNs produces *k* correct outputs.

Note that *U_cj_*(*z*, *l_c_*) can be obtained by using the recurrent expression:

(31) $$U_{cj}(z,l_{c})=U_{cj-1}(z,l_{c})\underset{+}{⊗}\left[r_{cm_{j}}z^{1}+(1-r_{cm_{j}})z^{0}\right]$$

According to its definition, *p_cj_*(*l_c_*) is the probability that the group of first *j* SNs produces *k_c_* correct outputs and the group of first *j* − 1 SNs produces *k_c_*− 1 correct outputs, given that *l_c_* SNs can be executed simultaneously. The coefficient $\pi_{jk_{c}}$ in polynomial *U_cj_*(*z*, *l_c_*) is equal to the conditional probability that the group of first *j* SNs produces *k_c_* correct outputs given that *l_c_* SNs can be executed simultaneously.

In order to let the coefficient $\pi_{jk_{c}}$ in polynomial *U_cj_*(*z*, *l_c_*) be equal to *p_cj_*(*l_c_*), the term with the exponent equal to *k_c_* should be removed from *U_cj_*_−1_(*z*, *l_c_*) before applying Equation (31) (excluding the combination in which *j* − 1 first SNs produce *k_c_* correct outputs while the *m_j_*-th SN fails).

If after the execution of *j* first SNs the number of correct outputs produced is *k* and *k* + *n_c_* − *j* < *k_c_* then the required number of correct outputs *k_c_* cannot be obtained even if all the *n_c_* − *j* subsequent SNs produce correct outputs. Therefore, the terms *π_jk_z^k^* with *k* < *k_c_* − *n_c_* + *j* can be removed from *U_cj_*(*z*, *l_c_*).

The above considerations lie at the base of Algorithm 2 for determining all of the probabilities *p_cj_*(*l_c_*) (*k_c_* ≤ *j* ≤ *n_c_*). Having the execution time distribution of each cluster, we can further evaluate that of the entire system, which is proposed in the next section.

#### 4.3.2. Evaluating the Execution Time Distribution of the Entire System

Having the pairs $<p_{cj}(l_{c}(x)),t_{cm_{j}}(l_{c}(x))>$ for each possible realization *l_c_*(*x*) of *L_c_* (1 ≤ *x* ≤ *h_c_*) and probabilities Pr{*L_c_* = *l_c_*(*x*)} = *Q_c_*(*x*), one can obtain the PMF of random execution time *T_c_* for each cluster by applying Equation (24). If the conditional PMF *p_cj_*(*l_c_*(*x*)), $t_{cm_{j}}(l_{c}(x))$ are represented by the u-function:

(32) $${\tilde{u}}_{c}(z,l_{c}(x))=\sum_{j=k_{c}}^{n_{c}}p_{cj}(l_{c}(x))z^{t_{cm_{j}}(l_{c}(x))}$$

then the u-function representing the PMF of the random value *T_c_* takes the form:

(33) $${\tilde{U}}_{c}(z)=\sum_{x=1}^{h_{c}}Q_{c}(x){\tilde{u}}_{c}(z,l_{c}(x))$$

Since the random system execution time *T* is equal to the sum of the execution time of all of the *C* clusters, one can obtain the u-function $\tilde{U}(z)$ representing the PMF of *T* as:

(34) $$\tilde{U}(z)=\underset{+}{⊗}\left({\tilde{U}}_{1}(z),\cdots ,{\tilde{U}}_{C}(z)\right)=\prod_{c=1}^{C}\left(\sum_{x=1}^{h_{c}}Q_{c}(x)\;{\tilde{u}}_{c}(z,l_{c}(x))\right)$$

#### 4.3.3. Evaluating the Different Clusters Consecutively Executed on the Same Hardware

Now consider the case where all of the clusters are consecutively executed on the same hardware consisting of *h* parallel identical modules with the availability *a*. The number of available parallel hardware modules *H* is random with PMF *Q*(*x*) = Pr{*H* = *x*}, 1 ≤ *x* ≤ *h*, defined in the same way as in Equation (27).

When *H* = *x*, the number of SNs that can be executed simultaneously in each cluster *c* is *l_c_*(*x*). The u-functions representing the PMF of the corresponding cluster execution time *T_c_* are ${\tilde{u}}_{c}(z,l_{c}(x))$ defined by Equation (32). The u-function $\hat{U}(z,x)$ representing the conditional PMF of the system execution time *T* (given the number of available hardware modules is *x*) can be obtained for any *x* (1 ≤ *x* ≤ *h*) as:

(35) $$\hat{U}(z,x)=\underset{+}{⊗}\left({\tilde{u}}_{1}(z,l_{1}(x)),\cdots ,{\tilde{u}}_{C}(z,l_{C}(x))\right)=\prod_{c=1}^{C}{\tilde{u}}_{c}(z,l_{c}(x))$$

Having the PMF of the random value *H*, we obtain the u-function $\tilde{U}(z)$ representing the PMF of *T* as:

(36) $$\tilde{U}(z)=\sum_{x=1}^{H}Q(x)\;\hat{U}(z,x)$$

#### 4.3.4. Optimizing the Structure of SCAs in WSNs

When a SCA with FT in WSNs is designed, one has to select SNs for each cluster and find the sequence of their execution in order to achieve the greatest system reliability subject to cost constraints. The SNs are selected from a list of the available products. Each SN can be characterized by its reliability, execution time, and cost. The total cost of the system is defined according to the cost of its SNs. For each SN, its cost may be a purchase cost (if the SN or its data observation is provided by a commercial service). It also may be a comprehensive cost based on the SN’s size, complexity, and performance.

Assume that *B_c_* functionally equivalent SNs are available for each cluster *c* and that the number *k_c_* of the SNs that should agree in each cluster is predetermined. The choice of the SNs and the sequence of their execution in each cluster determine the system’s entire reliability and performance.

The permutation x*_c_ of *B_c_* different integer numbers ranging from 1 to *B_c_* determines the order of the SN that can be used in cluster *c*. Let *y_cb_* = 1 if the SN *b* is selected to be included in cluster *c* and *y_cb_* = 0 otherwise. The binary vector ***y****_c_* = {*y_c_*_1_, …, $y_{cB_{c}}$} determines the subset of SNs selected for cluster *c*. Having the vectors x*_c_ and *y_c_* one can determine the execution order *x_c_* of the SNs selected by removing from x*_c_ any number *b* for which *y_cb_* = 0. The total number of SNs in cluster *c* (equal to the length of vector *y_c_* after removing the unchosen SNs) is determined as:

(37) $$n_{c}=\sum_{b=1}^{B_{c}}y_{cb}$$

The system structure optimization problem can now be formulated as find vectors ***x****_c_* for 1 ≤ *c* ≤ *C* that maximize *R*(*w*) subject to the cost constraint:

(38) $$\Omega =\sum_{c=1}^{C}\sum_{b\in x_{c}}\omega_{cb}\leq \Omega *$$

where *ω_cb_* is the cost of SN *b* used in cluster *c*, Ω is the entire system cost and Ω* is the MAX allowable system cost. Note that the length of vectors ***x****_c_* can vary depending on the number of SNs that are selected.

In order to encode the variable-length vectors *x_c_* in the GA using the constant length integer strings one can use (*B_c_ +* 1) − *length* strings containing permutations of numbers 1, …, *B_c_*, *B_c_* + 1. The numbers that appear before *B_c_ +* 1 determine the vector *x_c_*. For example, for *B_c_* = 5 the permutations (2,3,6,5,1,4) and (3,1,5,4,2,6) correspond to *x_c_* = (2,3) and *x_c_* = (3,1,5,4,2), respectively. Any possible vector *x_c_* can be represented by the corresponding integer substring containing the permutation of *B_c_* + 1 numbers. By combining *C* substrings corresponding to different clusters one obtains the integer string *a*, that encodes the entire system structure.

The encoding method is used in which the single permutation defines the sequences of the SNs selected in each of the *C* clusters. The solution encoding string is a permutation of $n=\sum_{c=1}^{C}\left(B_{c}+1\right)$ integer numbers ranging from 1 to *n*. Each number *j* belonging to the interval $\sum_{c=1}^{m-1}\left(B_{c}+1\right)+1\leq j\leq \sum_{c=1}^{m}\left(B_{c}+1\right)$ corresponds to SN $j-\sum_{c=1}^{m-1}\left(B_{c}+1\right)$ of cluster *m*. The relative order in which the numbers corresponding to the SNs of the same cluster appear in the string determines the structure of this cluster.

In order to examine the feasibility of our algorithm for SCAs with FT in WSNs, some experiments have been performed, which are presented in the next section.

## 5. Experiments and Analysis

Consider a SCA with FT in a WSN, which consists of five clusters running on fully available hardware. The parameters of the SNs that can be used in these clusters are described in Table 2. From this table, one can see that there are six SNs in cluster 1, five SNs in cluster 2, eight SNs in cluster 3, four SNs in cluster 4, and five SNs in cluster 5. This table contains the values of *L_c_* and *k_c_* for each cluster and the execution time *τ*, cost *c*, and reliability *r* for each sensor node.

**Table 2:** Parameters of clusters and sensor nodes.

No. of Cluster	*L_c_*	*k_c_*	Indices	No. of Sensor Nodes in Each Cluster							
				1	2	3	4	5	6	7	8
1	3	4	*τ*	17	12	9	32	10	55	-	-
			*c*	5	7	10	9	8	4	-	-
			*r*	0.82	0.80	0.97	0.92	0.94	0.88	-	-
2	3	3	*τ*	27	38	22	41	47	-	-	-
			*c*	10	9	12	4	7	-	-	-
			*r*	0.81	0.89	0.95	0.88	0.94	-	-	-
3	5	6	*τ*	17	22	36	25	15	39	29	43	
									
			*c*	9	2	14	7	10	8	15	13	
			*r*	0.91	0.80	0.96	0.88	0.93	0.95	0.97	0.97	
4	3	3	*τ*	7	5	10	22	-	-	-	-	
			*c*	5	8	9	10	-	-	-	-	
			*r*	0.75	0.85	0.93	0.97	-	-	-	-	
5	2	4	*τ*	25	15	13	27	48	-	-	-	
			*c*	4	8	12	7	10	-	-	-	
			*r*	0.87	0.85	0.96	0.90	0.98	-	-	-	

### 5.1. Experimental Environment

**Figure 4:**
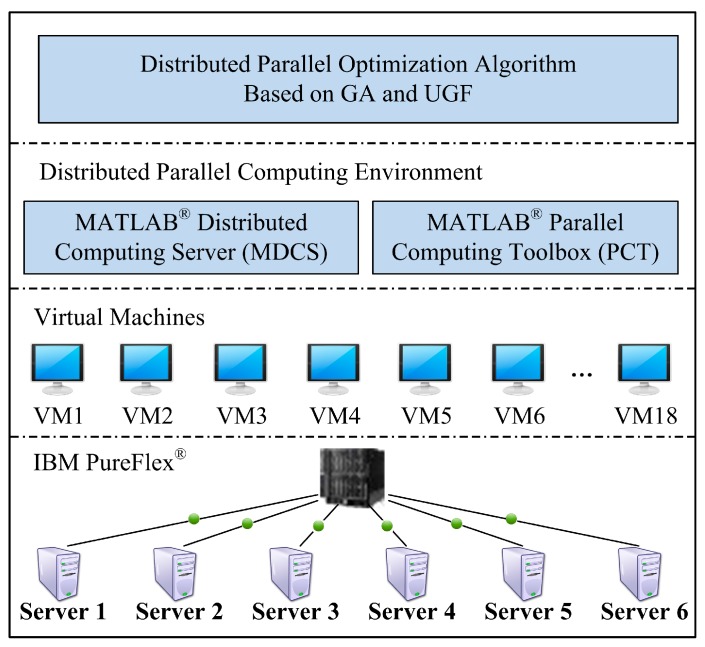
Cloud computing platform for the suggested algorithm execution in parallel.

In order to investigate the efficiency and performance of the suggested algorithm, we have developed a parallel GA program based on MATLAB^®^ Distributed Computing Server (MDCS) (The MathWorks, Inc., Natick, MA, USA) and Parallel Computing Toolbox (PCT) (The MathWorks, Inc., Natick, MA, USA). A cloud computing platform based on IBM PureFlex^®^ cluster with six blade servers was used for this in-depth experimental analysis. In this cloud computing platform, eighteen virtual machines have been built for searching optimal solution in parallel in our GA program, which is shown as Figure 4.

We built 16 parallel process nodes (named worker in PCT), 1 MDCS node, and 1 master node on 18 virtual machines. The 16 worker nodes undertake the parallel computing of the UGF of each SN, each cluster and the entire SCA, such as Algorithm 1 and Algorithm 2. The MDCS node is responsible for assigning computing tasks to each worker node and receiving their calculation results. The master node implements the establishment of the input data and the process control of the parallel computing.

Based on the above experiment platform, we compared and analyzed the dependencies among the optimal reliability, the given expected execution time, and the MAX allowable system cost, as well as the robustness of the suggested algorithm, which are described in the following sections.

### 5.2. Experimental Analysis

In order to investigate the change of reliability *R*(w*) of a SCA in WSNs along with the given expected execution time w* under some given cost constraints Ω*, a set of experiments was designed. In suggested GA algorithm, the population size is set to 500 chromosomes. The max generation is set to 100. The size of the optimal chromosome pool is dynamically increased from 0.5% to 5% of population size with the increase in the number of generations. The crossover probability is set to 0.7. The variation probability is set to 0.3. The penalty factor of reliability is set to 100. The number of repeated experiments is set to 30. A set of experiments were performed, which observe the searching process for the reliability function *R*(w*) along with along with the execution time *w* under the two given expected execution time (*i.e.*, w* = 250 and w* = 300) and the given cost constraints (*i.e.*, Ω* = 160, Ω* = 140, Ω* = 120 and Ω* = 100), respectively.

Given two expected execution time (w* = 250 and w* = 300), two sets of solutions were obtained using the suggested algorithm, which are described in Table 2. For each value of w*, four different solutions (*i.e.*, the optimal execution sequences of SNs), were obtained for the four given cost constraints (Ω* = 160, Ω* = 140, Ω* = 120 and Ω* = 100). The tables contain the minimal possible system execution time *T_min_*, maximal possible system execution time *T_max_*, the system cost Ω and reliability *R*(w*) for each solution, the expected conditional execution time $\tilde{\varepsilon }(\infty )$, and the corresponding execution sequences of the selected SNs.

Under each constant constraint on the MAX allowable system cost Ω*, the change trend of the total system cost Ω and the optimal reliability *R*(w*) was investigated along with the execution time *w*. Comparing the total system cost Ω and the reliability *R*(w*) of the optimal solutions corresponding to w* = 250 and w* = 300 in Table 2, it can be seen that the total system cost Ω and the reliability *R*(w*) of the optimal solution corresponding to w* = 300 is always equal or greater than ones corresponding to w* = 250 in the case of the same value of Ω*. 

Furthermore, under the given expected execution time w*, the change trend of the optimal reliability *R*(w*) was investigated along with the MAX allowable system cost Ω*. The optimal *R*(w*) has been found for Ω* changes from 100 to 160 with a constant incremental change of 20. Comparing the total system cost Ω and the reliability *R*(w*) of the optimal solutions corresponding to four different MAX allowable system costs in Table 3, it can be seen that the total system cost Ω and the reliability *R*(w*) of the optimal solution corresponding to larger Ω* is always equal or greater than ones corresponding to smaller Ω* in the case of the same value of *w*. From Table 2, it can also be seen that the system reliability gradually become greater along with the growth of the value of Ω*.

**Figure 5:**
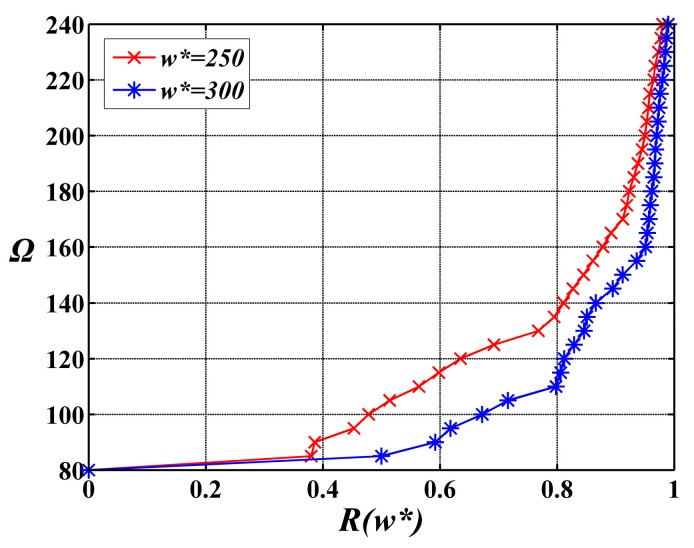
The cost-reliability curves with alterations in the cost from 80 to 240 under the two expected execution times w* = 250 and w* = 300 given by user.

Furthermore, the relationship between reliability and cost is investigated. The cost-reliability curves with alterations in the cost Ω from 80 to 240 under the two given expected execution time w* = 250 and w* = 300 by user are presented in Figure 5. Each point on these curves corresponds to the best solution obtained by the suggested algorithm. It can be seen that the greater the reliability level achieved, the greater the cost of further reliability improvement. In other words, for a greater reliability level, more SNs are need. From the designer’s perspective, he or she can intuitively find out the points which meet the requirements of reliability in Figure 5. Therefore, the corresponding cost can be found. On this basis, the decision on the reasonable quantity of SNs can be made. By this method, the structure of a WSN service application system can be further optimized under the condition satisfying the reliability requirements.

**Table 3:** Parameters of solutions obtained for w* = 250 and w* = 300.

w*	Ω*	Execution Sequence of SNs x*	*T_min_*	*T_max_*	Ω	$\tilde{\varepsilon }(\infty )$	*R*(250)
250	160	6435|352|316875|423|5317	181	289	160	197.25	0.901
	140	2316|345|426175|213|2451	173	274	127	215.68	0.847
	120	4612|415|432156|241|2341	218	257	118	239.73	0.764
	100	6152|452|852164|213|2541	199	238	97	248.41	0.692
300	160	6435|352|316875|423|3751	289	317	158	222.27	0.931
	140	3162|354|542761|231|2451	257	277	132	239.44	0.861
	120	1642|145|164325|241|4123	263	243	114	247.68	0.819
	100	2615|425|154268|213|5241	205	231	100	256.43	0.753

**Figure 6:**
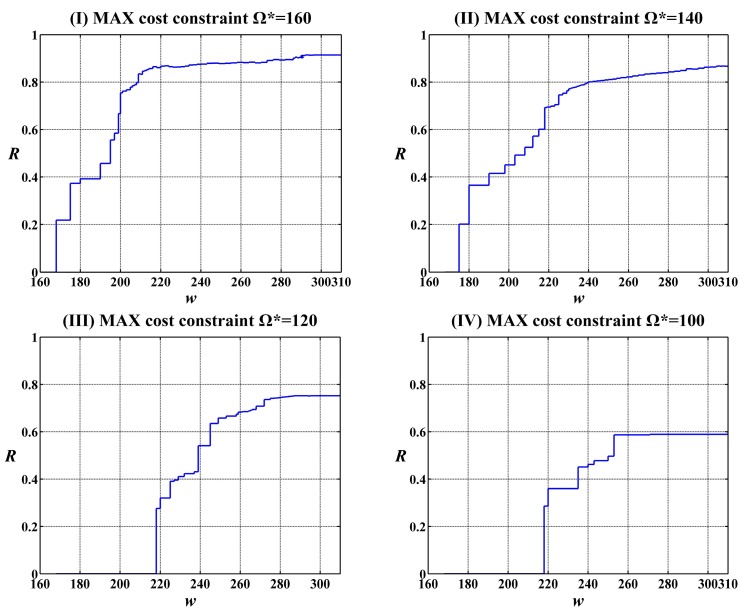
Change of the values of reliability function *R*(w*) along with the execution time *w* under the given expected execution time w* = 250.

**Figure 7:**
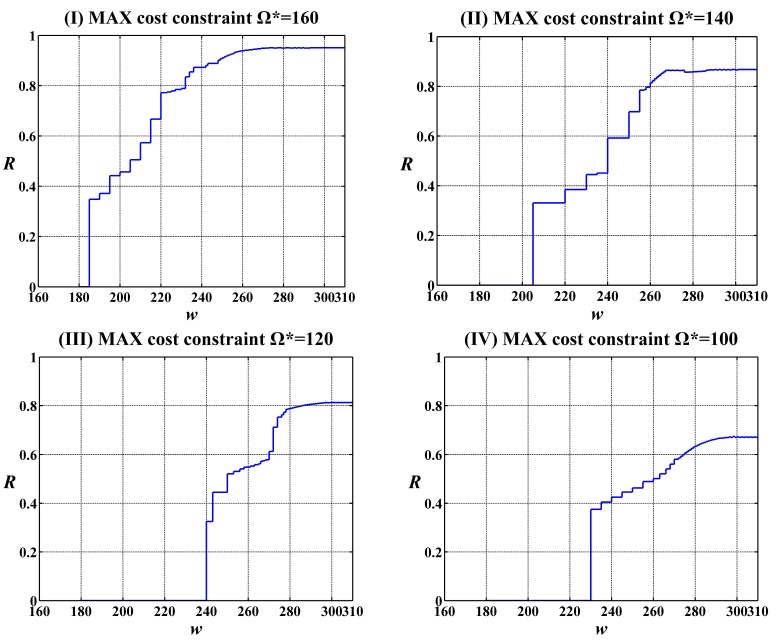
Change of the values of reliability function *R*(w*) along with the execution time *w* under the given expected execution time w* = 300.

In addition, under each constant constraint on the MAX allowable system cost Ω*, the change trend of the reliability function *R*(w*) was investigated along with the execution time *w*. The curves of the values of reliability function *R*(w*) for the execution time *w* change from 160 to 310 under four constraints on the MAX allowable system costs and two given expected execution time w* = 250 and w* = 300 by user are shown in Figure 6 and Figure 7, respectively. It can been seen that the values of reliability function *R*(w*) improve gradually along with the growth of *w*.

**Figure 8:**
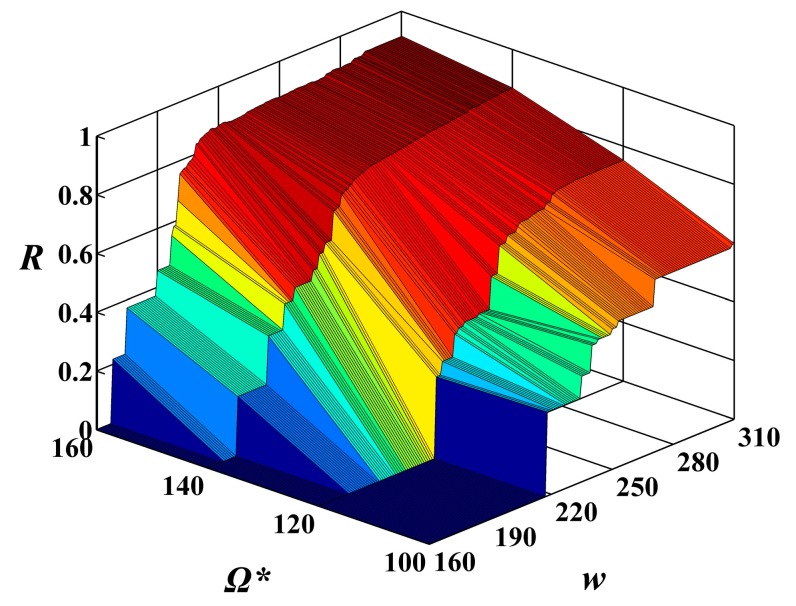
The values of reliability function *R*(w*) with alterations in the execution time *w* from 160 to 310 and in the given cost constraint Ω* from 100 to 160 under the given expected execution time w* = 250.

**Figure 9:**
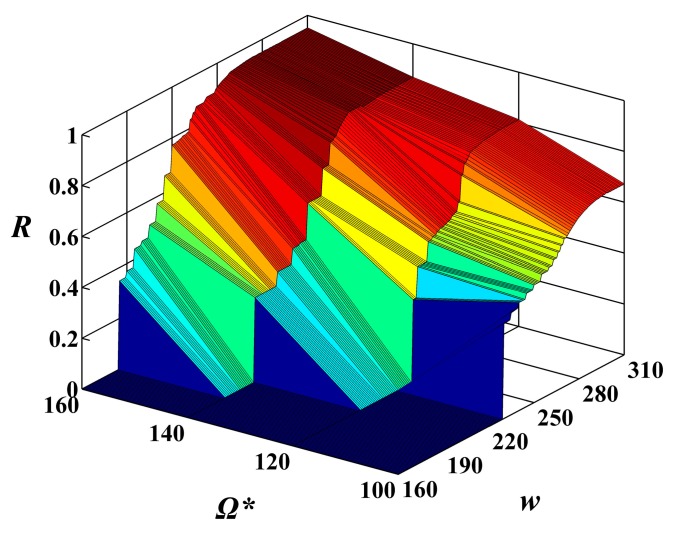
The values of reliability function *R*(w*) with alterations in the execution time *w* from 160 to 310 and in the given cost constraint Ω* from 100 to 160 under the given expected execution time w* = 300.

In order to investigate the relationship of the constraints on the MAX allowable system cost Ω* and the expected execution time w* in the combining effects on the system reliability of feasible solutions, the above experimental results are shown in the form of 3D image in Figure 8 and Figure 9, respectively. From these figures, one can see that the constraint on the MAX allowable system cost Ω* and the expected execution time w* influence the system reliability of feasible solutions, while the constraint on the MAX allowable system cost Ω* plays a more important role in the increase of the system reliability of feasible solutions than the expected execution time w*.

The above experimental analysis indicates that the selection of suitable Ω* and w* is helpful to improve the reliability of SCAs in WSNs and to cut down their cost. In the next section we present a distinct approach to selecting the most suitable Ω* and w* for the designers of SCAs in WSNs.

**Figure 10:**
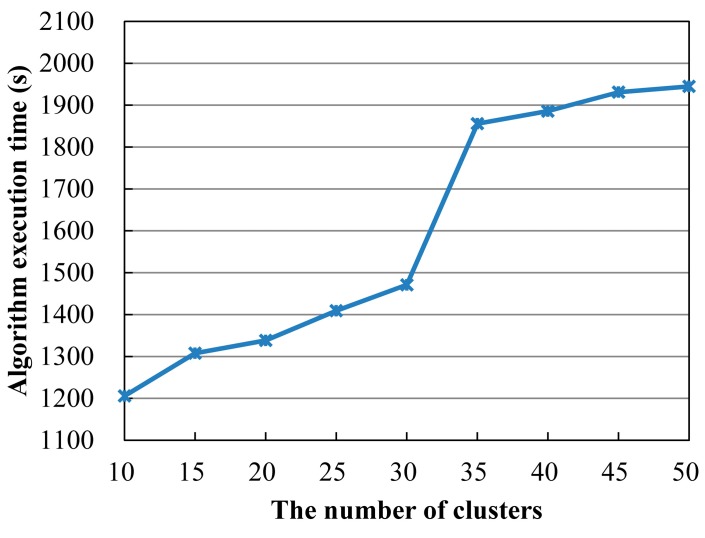
Algorithm execution time along with the number of clusters growing from 10 to 50.

In order to investigate the scalability of the proposed algorithms, we completed a series of experiments on the above cloud computing platform along with the number of clusters in a SCA system growing from 10 to 50 with a step growth of 5. Each cluster randomly contains 8 to 10 SNs. The *L_c_* of each cluster is set to a random number ranging from 4 to 8. The *K_c_* of each cluster is set to a random number from 6 to 8. The execution time *τ* of each SN is set to a random number from 10 to 50. The cost *c* of each SN is set to a random number from 3 to 10. The reliability *r* of each SN is set to a random number from 0.80 to 0.99. The parameters of GA program are set in the same as the experiment above. By inserting a pair of timers in the GA program, the exact algorithm execution time (not including data preparation time and task allocation time) is obtained. For each a number of clusters, we ran the GA program 20 times, and calculated the mean algorithm execution time for each a number of clusters. Figure 10 shows the changes of mean algorithm execution time along with the number of clusters increased from 10 to 50.

From Figure 10 one can see that the algorithm execution time gradually rises as the number of clusters is increased. In the two separate stages that the number of clusters increased from 10 to 30 and from 35 to 50, the algorithm execution time grows slowly (for ease of description, these two stages are referred to as the first Slow Growth Stage and the second Slow Growth Stage). However, in the stages that the number of clusters increased from 30 to 35, the algorithm execution time grows fast (for ease of description, this stage is referred to as the Fast Growth Stage). Through investigating the parallel task allocation by MDCS node, we found that in the first Slow Growth Stage the computing tasks of each worker node were not blocked in the task queue. It indicates that the computational load of each worker node is appropriate. All computing tasks assigned to every worker node can be fulfilled sequentially without waiting. However, in the Fast Growth Stage congestion began to appear in the task queues of worker nodes as the number of clusters increased sequentially. The computing tasks of worker nodes must wait for the completion of those tasks in front of them, which results a fast growth of the algorithm execution time. After this, with the continued increase in the number of clusters the execution of the algorithm has entered a new stage—the second Slow Growth Stage due to the load balancing generated by 16 worker nodes. Based on the above analysis, we can see from the two Slow Growth Stages that the proposed algorithm showed good scalability.

### 5.3. A Distinct Approach to Selecting the Most Suitable Ω* and w*

**Figure 11:**
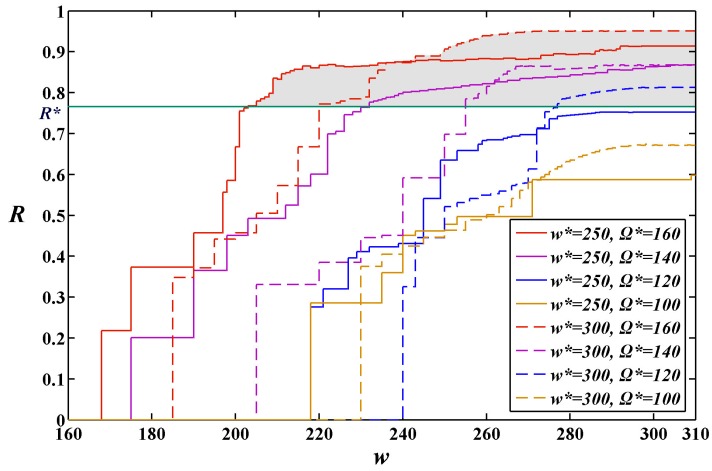
The values of reliability function *R*(w*) with changes in the execution time *w* from 160 to 310 under the given cost constraint on Ω* with changes from 100 to 160 and on the two given expected execution times (w* = 250 and w* = 300).

In order to help designers of SCAs in WSNs to select the most suitable Ω* and w*, the curves of the values of reliability function *R*(w*) under two constant cost constraints w* = 250 and w* = 300 for the expected execution time w* change from 160 to 310 are shown in Figure 11.

On the basis of the experimental analysis in the previous section, we present a distinct solution method for the designers of SCAs in WSNs to select the most suitable Ω* and w* based on Figure 9. For a reliability requirement from a user perspective, we can draw a horizontal auxiliary line according to the given value of reliability requirement R*. The intersection between the horizontal auxiliary line and the reliability curves forms multiple shadowed areas. The points falling into the shadow areas represent the feasible solutions subject to *R*(w*) ≥ R*. From Figure 9, one can see that not all curves of reliability function *R*(w*) intersect the horizontal auxiliary line. This indicates that only part of solutions meet the given reliability requirement in this example. Specifically, there are five sets of Ω* and w* suitable for the given reliability requirement, *i.e.*, (w* = 250 and Ω* = 160), (w* = 250 and Ω* = 140), (w* = 300 and Ω* = 160), (w* = 300 and Ω* = 140) and, (w* = 300 and Ω* = 120). Obviously, the set of Ω* and w* (w* = 300 and Ω* = 120) is the most suitable for the users who are more concerned about cost. On the contrary, the set of Ω* and w* (w* = 250 and Ω* = 140) is the most suitable for the users who are more concerned about system performance. In addition, one can see that the reliability corresponding to the set of Ω* and w* (w* = 300 and Ω* = 160) is higher than other when *w* > 240. Therefore, it is the most suitable for the users who are more concerned about system reliability.

Using the approach suggested above designers can easily find which sets of Ω* and w* can meet the reliability requirements of users. Furthermore, designers can easily find the most suitable set of Ω* and w* for different types of users.

**Figure 12:**
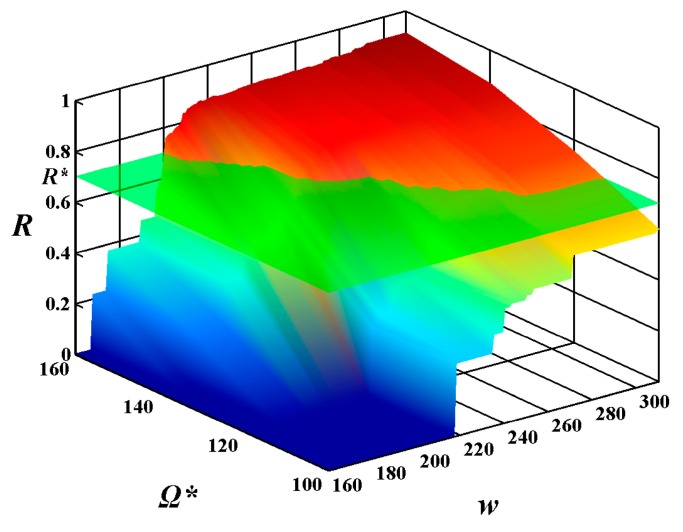
The selection for the most suitable Ω* and w* with alterations in the execution time *w* from 160 to 310 and in the given cost constraint Ω* from 100 to 160 under the given expected execution time w* = 250.

**Figure 13:**
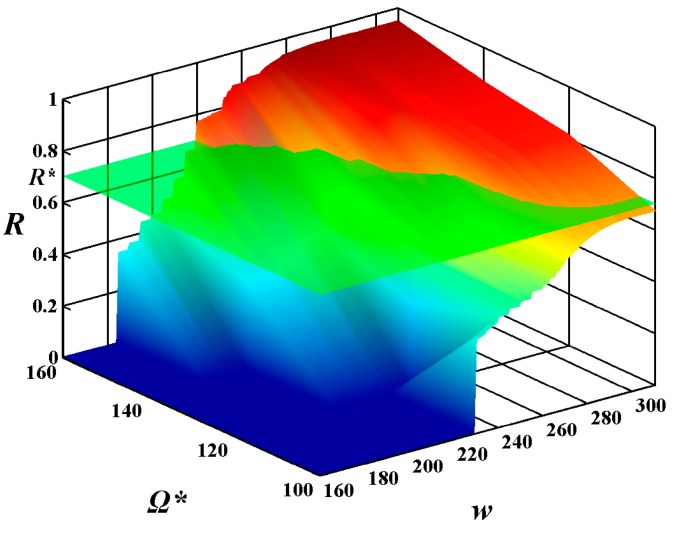
The selection for the most suitable Ω* and w* with alterations in the execution time *w* from 160 to 310 and in the given cost constraint Ω* from 100 to 160 under the given expected execution time w* = 300.

In order to better display the efficiency of the suggested algorithm, the selection for the most suitable Ω* and w* is shown in the form of a 3D image in Figure 12 and Figure 13, respectively. Unlike Figure 11, the reliability requirement R* is not a horizontal auxiliary line but rather an auxiliary plane. The auxiliary plane intersects the surface of the system reliability in Figure 8 and Figure 9, respectively. The most suitable Ω* and w* are located on the secant formed by the auxiliary plane and the surface of the system reliability. After carefully balancing the cost and the execution time, the designers can find which sets of Ω* and w* can meet the reliability requirements of users.

Generally, based on the most suitable set of Ω* and w* that are found by the suggested approach, the optimal structure of SCAs with fault-tolerant in WSNs, *i.e.*, the SNs in each cluster as well as their execution sequence, can be found using the suggested algorithms, which can provide as high as possible system reliability and performance under a given cost constraint proposed by users.

The suggested algorithms and approach presented in this paper can be easily realized by software. Furthermore, it has high enough efficiency because a fast algebraic procedure is used for finding the performance distribution of the entire WSN service system based on those of SNs on which the WSN service is running, therefore, it can also be used in online optimization situations.

## 6. Conclusions

Traditional reliability and performance optimization methods, such as the Markov model and state space analysis, have some defects such as being too time-consuming, facility for causing state space explosions and unsatisfactory assumptions of component execution independence, therefore they are inapplicable to the ever-changing SCAs in WSNs. In this paper, a novel reliability and performance optimization model based on MSS for WSN services systems is proposed, which eliminates the limitation for component execution independence, and fits better the actual execution of SCAs in WSNs. Based on UGF and GA, an efficient optimization algorithm for the reliability and performance of SCAs with fault tolerance in WSNs is presented, which eliminates the risk of state space explosion, and provides the system with as high reliability and performance as possible under a given cost constraint proposed by users. The suggested algorithms and approach presented in this paper can be used in the optimization for the reliability and performance of SCAs in WSNs both at the design and the execution phase.
